# Circulating Placental Alkaline Phosphatase Expressing Exosomes in Maternal Blood Showed Temporal Regulation of Placental Genes

**DOI:** 10.3389/fmed.2021.758971

**Published:** 2021-12-24

**Authors:** Arshiya Parveen, Suman Mishra, Medha Srivastava, Dharmendra K. Chaudhary, Deepa Kapoor, Amrit Gupta, Swasti Tiwari

**Affiliations:** ^1^Department of Molecular Medicine & Biotechnology, Sanjay Gandhi Post Graduate Institute of Medical Sciences, Lucknow, India; ^2^General Hospital, Sanjay Gandhi Post Graduate Institute of Medical Sciences, Lucknow, India; ^3^Department of Maternal & Reproductive Health, Sanjay Gandhi Post Graduate Institute of Medical Sciences, Lucknow, India

**Keywords:** maternal health, placenta, liquid biopsy, extracellular vesicles, antenatal care

## Abstract

**Background:** Analysis of placental genes could unravel maternal-fetal complications. However, inaccessibility to placental tissue during early pregnancy has limited this effort. We tested if exosomes (Exo) released by human placenta in the maternal circulation harbor crucial placental genes.

**Methods:** Placental alkaline phosphate positive exosomes (ExoPLAP) were enriched from maternal blood collected at the following gestational weeks; 6–8th (T1), 12–14th (T2), 20–24th (T3), and 28th−32nd (T4). Nanotracking analysis, electron microscopy, dynamic light scattering, and immunoblotting were used for characterization. We used microarray for transcriptome and quantitative PCR (qPCR) for gene analysis in ExoPLAP.

**Results:** Physical characterization and presence of CD63 and CD9 proteins confirmed the successful ExoPLAP enrichment. Four of the selected 36 placental genes did not amplify in ExoPLAP, while 32 showed regulations (*n* = 3–8/time point). Most genes in ExoPLAP showed significantly lower expression at T2–T4, relative to T1 (*p* < 0.05), such as *NOS3, TNFSF10, OR5H6, APOL3*, and *NEDD4L*. In contrast, genes, such as *ATF6, NEDD1*, and *IGF2*, had significantly higher expression at T2–T4 relative to T1. Unbiased gene profiling by microarray also confirmed expression of above genes in ExoPLAP-transcriptome. In addition, repeated measure ANOVA showed a significant change in the ExoPLAP transcriptome from T2 to T4 (*n* = 5/time point).

**Conclusion:** Placental alkaline phosphate positive exosomes transcriptome changed with gestational age advancement in healthy women. The transcriptome expressed crucial placental genes involved in early embryonic development, such as actin cytoskeleton organization, appropriate cell positioning, DNA replication, and B-cell regulation for protecting mammalian fetuses from rejection. Thus, ExoPLAP in maternal blood could be a promising source to study the placental genes regulation for non-invasive monitoring of placental health.

## Introduction

Placental development is an exceptionally coordinated cycle directed by various variables, such as hormones, development factors, protein kinases, growth factors, protein kinases, transcription factors, gap junction proteins, and intracellular proteases (1). Expression analyses on intrauterine growth restriction (IUGR)-associated placenta have provided further insight into the role of genes associated with oxidative stress and immune tolerance in cause or response to the development of growth phenotypes (2). Accordingly, the power of placental analysis to unravel maternal-fetal complications is well acknowledged. However, the limited material availability has seriously affected our understanding of the placental response or adaptation during early pregnancy. Thus, the clinical management of the two biggest challenges in antenatal care, prematurity, and pre-eclampsia, is still a challenge. In the last decade, organoids have emerged as novel models for biomedical research. They are small self-organized 3D tissue cultures derived from stem cells that mimic tissue type, such as blastoid, endometrium, and trophoblast tissues. They provide useful models for studying the process, such as embryo implantation, female reproductive tract, and disease modeling. At present, the main ECM used in the endometrium organoid culture is matrigel to facilitate organoids in fully mimicking their counterparts *in vivo* tissues. In addition, it is known that the present endometrium organoid culture mainly pays attention to epithelial cells, but focuses less on other types of cells, such as stromal cells, vascular endothelial cells, and immune cells, which are critical for disease development (3).

In the circulation, tissue-derived nanovesicles (aka Exo) have gained substantial research attention as a liquid biopsy approach. They are different than microvesicles which are found in blood circulation both, in terms of size and biogenesis. Size of the microvesicles ranges between 0.1 and 1.0 μm, they are shed by the outward blebbing of the plasma membrane. While Exo are spherical lipid-bilayer vesicles of 30–100 nm diameter, secreted from cells into the extracellular environment upon the fusion of multivesicular bodies containing intraluminal vesicles with the plasma membrane (4, 5). The Exo released by the human placenta in the maternal circulation can be differentiated by placental-specific alkaline phosphatase (PLAP) protein on their surface (6). Moreover, the numbers of these PLAP-expressing Exo in maternal blood correlated nicely with increased gestation periods across normal pregnancy (6, 7). Their numbers, however, were found to vary in mothers with pregnancy-related issues, such as gestational diabetes, pre-eclampsia, or suboptimal fetal growth relative to a healthy pregnancy (7–9). These PLAP+ve Exo (ExoPLAP) have been demonstrated to exhibit immunomodulatory properties (5). Exo obtained from placental perfusate or extravillous cells have also been studied (6, 10, 11).

Together, these studies suggest that ExoPLAP could reflect the placental tissue response or adaptation during early pregnancy. However, studies analyzing placental-derived Exo to monitor change in placental gene regulation during healthy pregnancy are lacking. We isolated ExoPLAP from maternal blood samples, collected at the following gestational weeks; 6–8th (first trimester), 12–14th (early-second trimester), 20–24th (late second trimester), and 28th−32nd (mid-third trimester) to study the expression and regulation of a few placental gene, manually curated from the literature. Additionally, an unbiased gene profiling was performed to further confirm the expression of these genes in the ExoPLAP transcriptome.

## Materials and Methods

### Sample Collection and Biochemical Screening

A prospective cohort of pregnant women, who visited the Obstetrics and Gynecology department for routine antenatal care were enrolled after written informed consent. The studies involving human participants were reviewed and approved by the Human Ethics Committee of Sanjay Gandhi Postgraduate Institute of Medical Sciences (Ref. no: IEC-2017-9-EMP-95, dated September 16, 2019). The women were followed during their periodic antenatal visits. Gestational age was estimated based on the first day of their last menstrual period and confirmed by transvaginal ultrasound at the recruitment (i.e., ~6–8 weeks). All women included in this study were free of apparent infections within the uterus or amniotic cavity, and all pregnancies were singletons. Obstetrical history and physical findings were recorded regarding previous spontaneous abortions, previous pregnancies, hypertension, gestational diabetes, and preeclampsia to confirm healthy pregnancy.

Peripheral venous blood sample (5 ml) was taken at the following gestation weeks, 6–10th (T1), 12–14th (T2), 20–24th (T3), and 28th−32nd (T4). Samples were collected in ethylene diamine tetraacetic acid (EDTA)-treated tubes (BD Vacutainer® Plus plastic plasma tube) and plain tubes (BD Vacutainer® Plus, NJ, USA), from which plasma and serum (for biochemical analysis) were obtained respectively by centrifugation at 3,000 rpm at 4°C for 10 min and stored in aliquots at −80°C until further processing. Samples collected at different gestation weeks were used for reverse transcription PCR (RT-PCR) (*n* = 3–8/time point). For transcriptome profiling ExoPLAP from longitudinally collected maternal samples from five different women (*n* = 5/time point) were used.

### Biochemical Screening for Aneuploidies

Serum levels of free beta-human chorionic gonadotrophin (beta-hCG) and pregnancy-associated plasma protein A (PAPP-A) hormones were estimated in maternal blood collected between 10th and 13th gestation weeks. Following hormones were estimated between 15th and 20th gestation weeks; Alpha-fetoprotein (AFP), Human chorionic gonadotropin (hCG), Inhibin A, and Unconjugated Estriol (UE). All the hormones were estimated by a chemiluminescence based assay (Immulite 1000, M/s Siemens Ltd., USA). Prisca software was used to calculate the risk for aneuploidies (Trisomy 13, 18, and 21) and risk for neural tube defects. For risk calculation, data of the ultrasound findings examining the nuchal translucency, nasal bone, and crown rump length were used in addition to biochemical estimations.

### Exosome Isolation and Characterization

First the plasma Exo were isolated using differential centrifugation as described by us previously (12–15). The pellet containing plasma Exo was then incubated with fluorescein isothiocyanate (FITC) conjugated anti-Placental alkaline phosphatase antibody for ExoPLAP enrichment for capture. After the incubation, the samples were ultracentrifuged, washed, and incubated with anti-FITC conjugated magnetic microbeads. The bead-antibody-Exo complex was separated through the appropriate column, followed by washing through centrifugation of the eluted complex. The enriched pellet was dissolved in 30 μl buffer. Blood from women without placenta (non-pregnant women) was used to demonstrate the specificity of the enrichment process.

### Nanotracking Analysis (NTA) and Dynamic Light Scattering Analysis

Size distribution and concentration of Exo were analyzed by nanoparticle tracking analysis (NTA) using the NanoSight NS300 (Malvern Instruments Ltd., UK) at the Central Analytical Research Facility of the Indian Institute of Toxicological Research, Lucknow, according to the instruction of manufacturer. In addition, dynamic light scattering (DLS) using a Nano Zetasizer (Malvern Instruments Ltd., UK) was performed as per the manufacturers protocol. The Gaussian fitting, mean value, and SD were calculated and compared using Origin Pro 9.0.0 (Origin Lab Corp, MA, USA).

### Transmission Electron Microscopy (TEM)

The ExoPLAP samples were prepared by dissolving in 2.5% (w/v) glutaraldehyde in cacodylate buffer. The prepared samples were then applied to a continuous carbon grid and negatively stained with 2% (w/v) uranyl acetate. The samples were examined by transmission electron microscope in the Central Analytical Research Facility of the Indian Institute of Toxicological Research, Lucknow, India.

### Immunoblotting

Exosomal protein samples were subjected to immunoblotting as described by us previously (12, 14). The immunoblotting was performed using antibodies against CD63, CD9, and PLAP (Abcam). Images were acquired on a ChemiDoc imaging system (Universal Hood III, BIO-RAD, CA, USA).

### RNA Extraction and Real-Time Quantitative PCR

RNA from ExoPLAP samples were isolated by RNAeasy kit (Qiagen India Pvt. Ltd, Delhi, India) as per manufacturer protocol. The quantity was checked by Nanodrop ND2000 (Thermo Fisher Scientific, Pittsburgh, PA, USA). For real time quantitative PCR (qRT-PCR), *in vitro* transcribed RNA was used. *In vitro* transcription was performed as described by us previously (16). In brief, *in vitro* transcribed RNA (100 ng) was subjected to cDNA synthesis using a high-capacity cDNA reverse transcription kit (Applied BioSystems, MA. USA). Relative gene expression was estimated by qPCR SYBR green PCR master mix (Takara Bio., Japan). Fold expression was calculated by 2–ΔCT method using 18S rRNA as an endogenous control. Supplementary Table 1 shows the sequences of the primers used.

### Microarray Analysis

RNA from ExoPLAP were subjected to microarray analysis using the instruction of manufacturer (Thermo Fisher Scientific). Transcriptome Analysis Console (version 4.0.0.25, Applied Biosystems) was used for the screening of differentially expressed genes among the three-gestational time points. The output files (.CEL) were analyzed by repeated measure ANOVA method of gene expression analysis in the settings. Repeated measures applied on each sample IDs, background correction, quantile, normalization, description, and log2 value conversion were done using the RMA+DABG algorithm. The principal component analysis (PCA) based on limma R/Bioconductor software package for integrative analysis of large-scale gene expression data was performed to assess the total similarities and disparities among the samples at the three time points (T2–T4) in the log-transformed expression ratios of genes. Minimum 50% of biological replicates displayed gene strength greater than their equivalent local background, the genes were constructed as expressed.

### Statistical Analysis

For statistical analysis, GraphPad Prism 8 was used. The significance of the mean was calculated using one-way ANOVA, followed by pair-wise comparison with T1; *p* < 0.05 was considered significant. The statistical analyses of the microarray were performed by Transcriptome Analysis Console software. Eventually, all statistically tested genes were filtered to acquire the significant ones for the differential gene expression analysis and formation of hierarchical clustering with a cut-off false discovery rate (FDR) *F*-test of < 0.01.

## Results

### Characterization of ExoPLAP From Pregnant Women With Healthy Pregnancy

Placental alkaline phosphate positive exosomes were isolated from the plasma samples of pregnant women (*n* = 10) at the following mean gestational weeks; 8 (T1), 14 (T2), 22 (T3), and 31 weeks (T4). Maternal serum biochemistry was done to assess any risk for fetal aneuploidies in the study participants (Table 1).

**Table 1:** Maternal serum biochemistry and demographics.

	**Mean (SD)**	**Healthy reference range**
**Risk for aneuploidies**		
Gestational age at sample collection for 1–4 parameters (weeks)	17.9 (1.72)	14–22^+6^
1. Alpha-fetoprotein (AFP) (ng/ml)	52.45 (19.29)	5.4–501
2. Unconjugated Estriol (UE3) (ng/ml)	1.145 (0.62)	0–11
3. Human chorionic gonadotropin (hCG) (mlU/ml)	20,496.7 (8,984.63)	2,223–200,230 varies with GA(14–22)
4. Inhibin A (Inh-A) (pg/ml)	287.705 (137.69)	236.78–373.33 varies with GA(14–22)
Gestational age at sample collection and ultrasound for 5–8 parameters (weeks)	12 (0)	10–13
5. Pregnancy-associated plasma protein A (PAPP-A) (mlU/ml)	4.57 (2.27)	0.1–32.3
6. Free beta-human chorionic gonadotrophin fb-hCG (ng/ml)	11.31 (13.56)	5.6–388.7
7. Crown rump length by ultrasound	62.7 (0.72)	24–84
8. Nuchal translucency MoM by ultrasound	0.68 (0.190)	
Risk for trisomy 18	<1:10,000	<1:100
Risk for trisomy 21	<1:10,000	<1:250
Risk for neural tube defects (NTD) (MoM AFP)	0.922 (0.33)	AFP MoM <2.5
**Demographics**		
Age at the time of enrolment (yrs)	28.3 (5.18)	
Gestational week at the time of delivery (weeks)	37.16 (0.75)	
Gravidity	1.83 (0.98)	
Parity	1.5 (0.54)	

*Data are presented as mean (SD)*.

**Figure 1:**
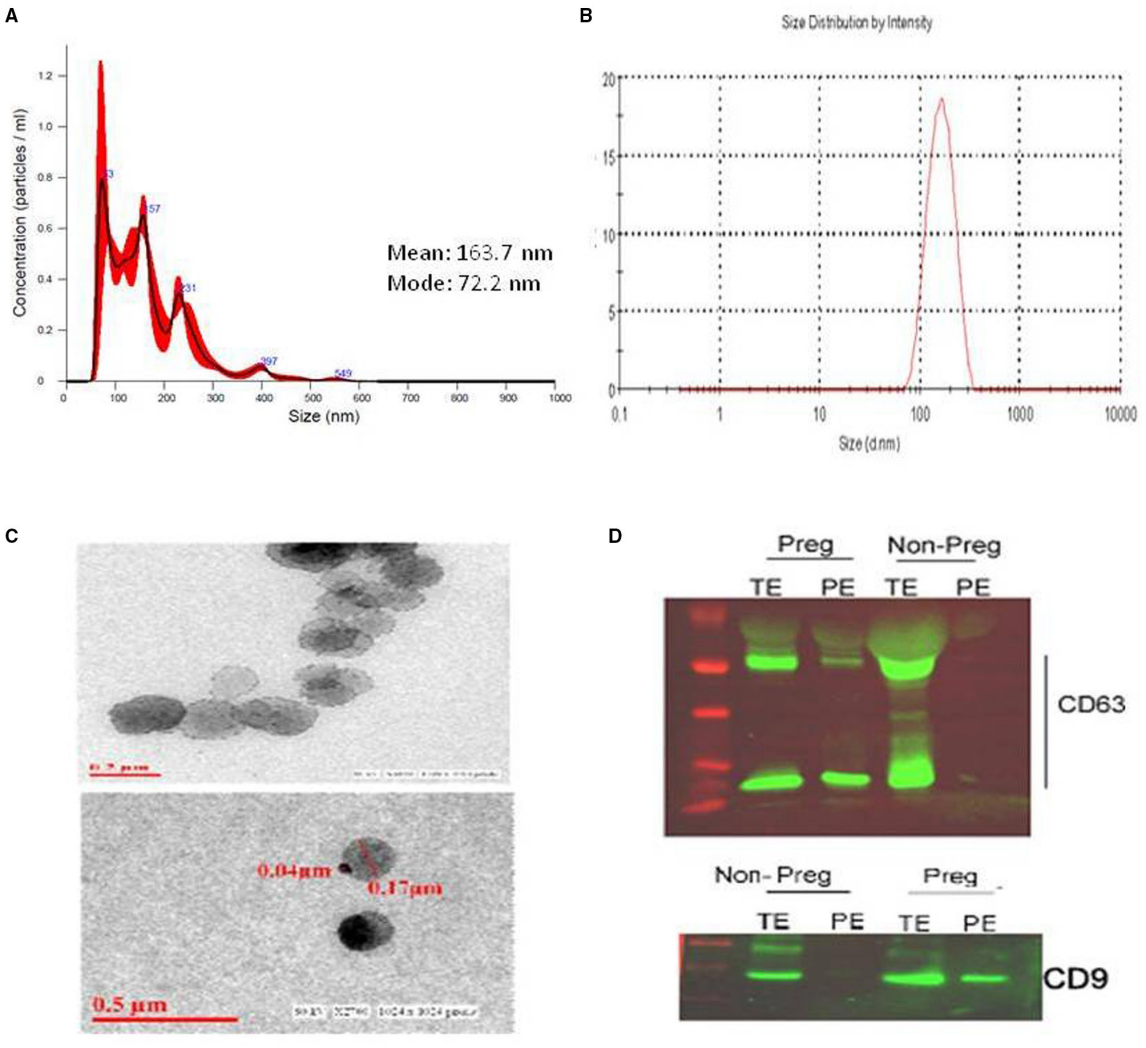
Isolation of placental alkaline phosphate positive exosomes (ExoPLAP) from maternal plasma. Size distribution and concentration of the exosomes as analyzed by **(A)** nanoparticle tracking analysis (NTA) and **(B)** dynamic light scattering **(C)** transmission electron micrograph of ExoPLAP-Ab complex captured on anti FITC-coated magnetic beads. **(D)** Immunoblot for CD63 and CD9 specific protein band in total plasma exosomes (TE), and in samples enriched from TE using ant-PLAP antibody (PE) in pregnant women (Preg) and non-pregnant women (Non-Preg) women. Full image of blot is submitted as Supplementary Material.

The electron microscopy and Dynamic Light Scattering analysis confirmed the expected physical characteristic of the ExoPLAP (Figures 1B,C). In addition, NTA, demonstrating the exosome sizes ranging from 100 to 200 nm in diameter is shown in (Figure 1A). Immunoblotting showed the presence of protein bands specific for exosomal-marker protein, CD63, and CD9 (second lane of Figure 1D) in the ExoPLAP samples collected from blood of pregnant women. However, the absence of CD63 and CD9 proteins bands in the last lane of Figure 1C indicate that the anti-PLAP antibody failed to enrich any ExoPLAP from the total exosome collected from women without placenta (non-pregnant women), this confirms the specificity of ExoPLAP enrichment method.

### Regulation of Pregnancy-Associated Genes in the ExoPLAP During Healthy Pregnancy

**Figure 2:**
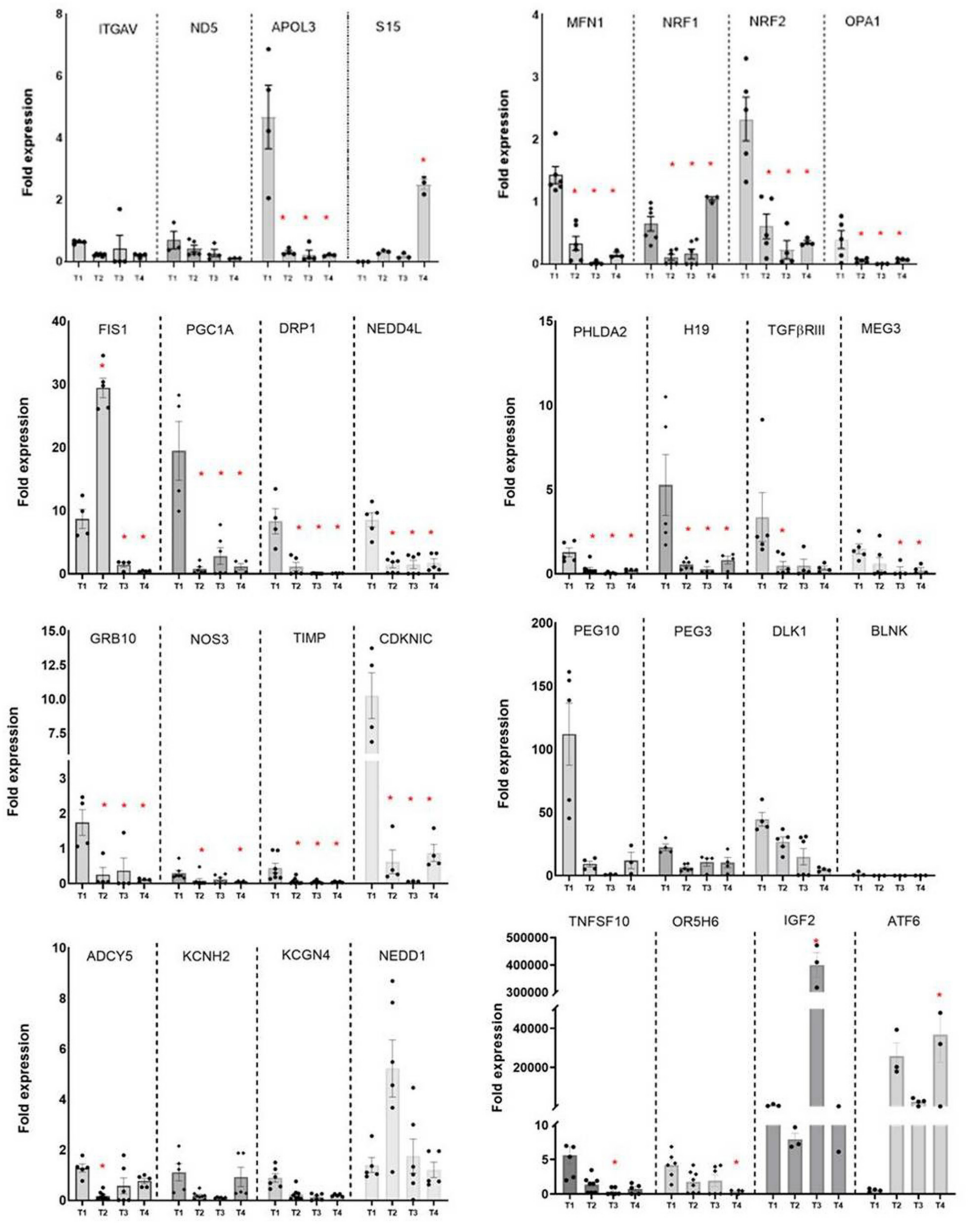
The mRNA expression of genes analyzed in the ExoPLAP in healthy pregnancy. Scatter plots with bar graph to show the fold expression of genes at the following mean gestational weeks, 8th (T1), 14th (T2), 22nd (T3), and 31st (T4). The dots in the scatter plot represents individual values, and bar graph shows mean ± SEM at each gestational week. Fold expression was estimated by quantitative PCR (qPCR) using calculated by 2–ΔCT method using 18S rRNA as an endogenous control. *p < 0.05 was considered significant by One-way ANOVA followed by Dunnett's *post-hoc* test for comparisons relative to T1 (*n* = 3–8/gestational week).

The expression of 36 pregnancy-associated genes, selected from the literature, was analyzed in the ExoPLAP at four key gestational time points (*n* = 3–8/time point). Four genes, i.e., *GNAS, IL-10, IL-13*, and *ORC5* did not amplify in these samples, however, 20 out of remaining 32 genes had higher transcript abundance in the ExoPLAP at the first trimester (T1) of pregnancy, including eight imprinted genes. The imprinted genes include *PHLDA2, DLK1, MEG3, PEG3, PEG10, GRB10, H19*, and *CDKNIC* (Figure 2), these had significantly lower expression at the second or third trimester (T2–T4); relative to T1, (*p* <0.05). Unlike these, the ninth imprinted gene analyzed in our study, *IGF2*, had significantly higher transcript abundance at the T3 time point, relative to T1 (Figure 2).

In addition, 19 other selected genes, which follow Mendelian inheritance, showed a trend similar to those of the eight imprinted genes except *NRF1, NOS3, DRP1*, and *ITGAV* genes (Figure 2). *NRF1* transcript levels had increased at T4 more than its level at T1 (*p* < 0.05). While for *NOS3*, the *DRP1* genes had significantly lower expression at second and third trimester time points (T2, T3, and T4) relative to the first trimester (T1). The transcript levels were lower at the second trimester time points for the rest of the genes and remained lower at T4, relative to T1. These include *PGC1A, ND5, NRF2, MFN1, OPA1, KCNG4, TIMP, TNFSF10, KCNH2, OR5H6, APOL3, NEDD4L, TGF*β*RIII*, and *BLNK*. Nine of these genes are mitochondrial metabolic genes, such as, *PGC1*α*, MFN1, NRF2, OPA1, DRP1*, and *ND5* (Figure 2).

On the other hand, the mRNA levels of the remaining five genes, such as *ATF6, NEDD1, S15*, and *FIS1*, had lower expression at T1, relative to T2 (*p* < 0.05, Figure 2). *ATF6* had the higher expression at T4, relative to T1.

### Change in Transcriptome Profile of the ExoPLAP With Pregnancy Progression

**Figure 3:**
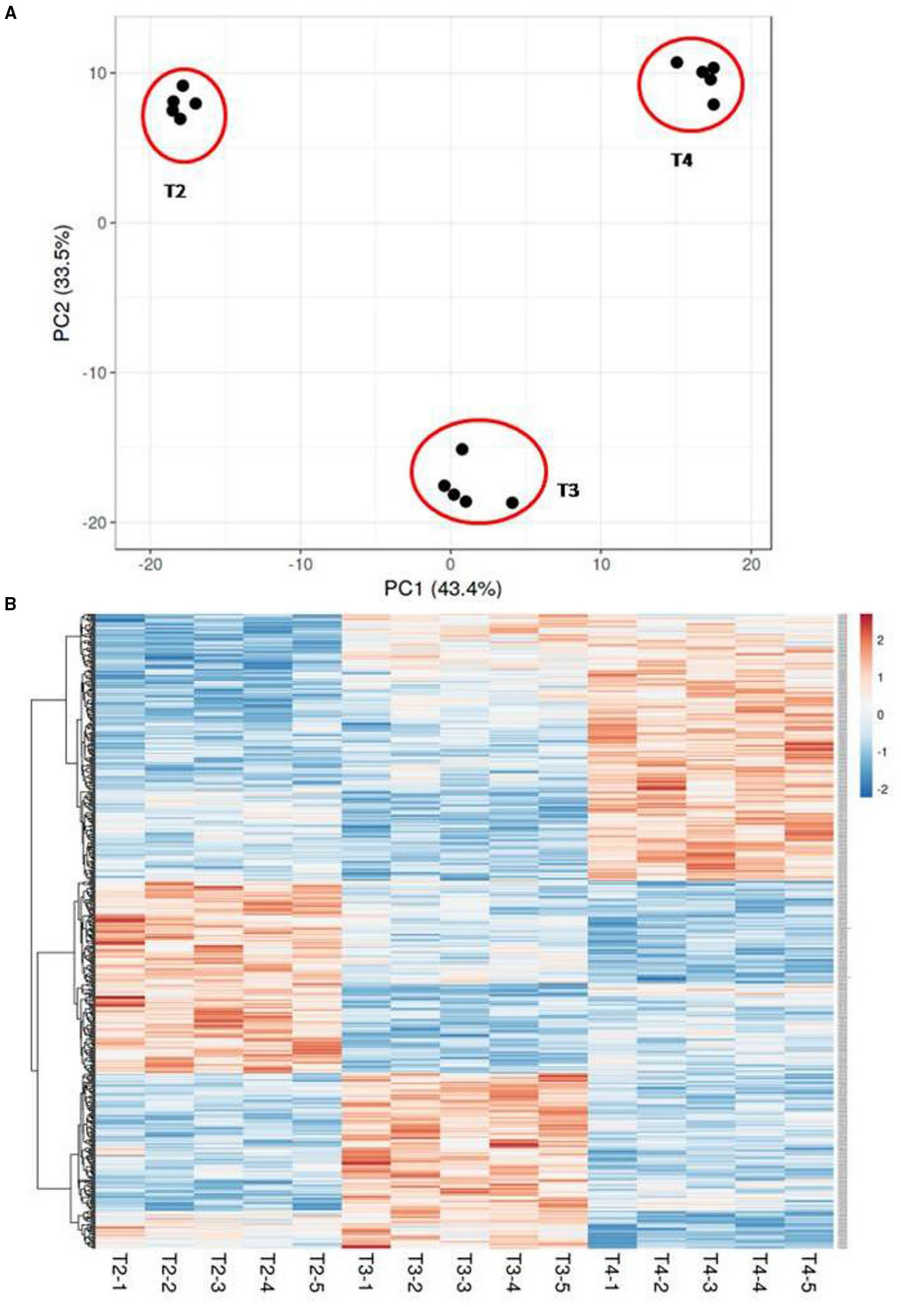
Change in the ExoPLAP-transcriptome with gestational age advancement in healthy pregnancy. **(A)** Principal components analysis (PCA) shows a significant distinction among all samples and indicated a clear separation among the three gestational time points, (mean gestational age, 14th (T2), 21st (T3), and 32nd (T4) weeks. **(B)** Heat map representing expression profiling of identified differentially regulated genes (*p* < 0.01) among the three different gestational time points by repeated measure ANOVA (*n* = 5/gestation week).

Transcriptome profiling of ExoPLAP was performed at T2, T3, and T4 time points using longitudinally collected samples from 5 different women (*n* = 5/time point). All the 36 selected genes were found to express in the transcriptome of ExoPLAP. The PCA for the overall structure of the analyzed dataset indicated a clear separation among three different gestational time points (T2, T3, and T4). There was a variance of 43.4% using component 1 (PCA1), and 33.5% using component 2 (PCA2) (Figure 3A). The sample from the same gestational time point lies in proximity to each other. Analysis showed that relative to the T1 time point, 4,590 genes were differentially regulated at T2 time points, respectively (*F*-test ≤ 0.05, Figure 3B). This set of genes included both assigned and unassigned genes. Figure 3B shows the heat map with hierarchical cluster analysis of the gene expression of these 502 genes (*p* ≤ 0.01). According to their site of occurrence, all the gene sets were further categorized.

## Discussion

The placenta plays a pivotal role in bringing out maternal physiological changes and fetal development during pregnancy. In this study, we tested if circulating ExoPLAP in the maternal blood could be used to study the temporal changes in placental gene regulation during pregnancy. We analyzed the expression and regulation of a few selected pregnancy-associated placental genes in ExoPLAP at key gestational weeks. The expression of the literature-selected genes in the ExoPLAP transcriptome was confirmed by an unbiased gene profiling. Besides, a clear change in the ExoPLAP transcriptome profile was noticed among the three gestational time points T2, T3, and T4.

Maternal serum biochemistry indicated low/no risk for fetal aneuploidies in the study participants. In addition, maternal-fetal outcomes further confirmed that the samples studied were from healthy pregnancies.

The 36 pregnancy-associated genes selected in our study were curated from the literature based on their reported role in early pregnancy progression. These genes are shown to have a key role in signal transduction, cell communication, apoptosis, nucleic acid metabolism, metabolism, energy pathway, ion transport, mitochondrial organization, and biogenesis. Furthermore, 32 genes (of 36) showed a good amount of expression in ExoPLAP, while four of the selected genes could not be amplified, such as *GNAS, IL-10, IL-13*, and *ORC5*. Most of the amplified genes were found to have a higher abundance in the first trimester ExoPLAP than the rest of the time points. These include *OR5H6* (Olfactory receptor family 5-member gene 6), *ITGAV* (Alpha V integrins), *BLNK* (B-linker protein), Apolipoprotein L3 (*APOL3*), *TRAIL* (TNF-related apoptosis-inducing ligand), and *ND5* (NADH-ubiquinone oxidoreductase chain 5). *OR5H6* has a role in appropriate cell positioning, an essential pathway/process during human embryogenesis in the first and early second trimesters (17). *OR5H6* may help to facilitate appropriate cell positioning and cell recognition during human embryogenesis (18, 19).

Besides, BLNK, a required component for B-cell development (18), has been suggested to regulate the developing maternal immune response of conceptus and could help protect a mammalian fetus bearing paternal alloantigens from rejection (20). Animal studies showed the role of *BLNK* in regulating B-cell function during conceptus attachment to the uterine endometrium. The other gene in this cluster is *ITGAV*, which plays an essential role during early pregnancy in the actin cytoskeleton and DNA replication (21, 22). It may be crucial for uterine receptivity in early pregnancy and during labor to facilitate the required syncytium formation (23, 24).

Angiogenesis is another important event during early placental development. A hypoxic environment in the early first trimester of pregnancy favors angiogenesis in the placenta. *APOL3* mRNA had a higher abundance in the T1 ExoPLAP relative to T2 and T3 time points. APOL3, a regulator of angiogenesis and endothelial tube formation, may act *via* ERK1/2, FAK, and Akt signaling (25). It may have a role in the early onset of preeclampsia (26).

With the progression of pregnancy toward the second trimester, extensive vascularization and placental mass expansion become crucial. During this time, the fetal vessels undergo extensive branching for placental development to meet the growing needs of the fetus. However, avoiding vascular complications becomes necessary due to excessive endoplasmic reticulum (ER) stress in the placenta (27). Hence, a lower expression of the NOS3 and potassium voltage (Kv) gated channel genes in the ExoPLAP may indicate a feedback response of the placenta to avoid such vascular complications (28). Nevertheless, altered expression of Kv may have an association with preeclampsia (29), intrauterine fetal death (30), genetic predisposition like sudden infant death syndrome (31), and congenital heart disease (32). Thus, we anticipate a gradual decrease in *NOS3, KCNH2*, and *KCNG4* gene expressions in the second trimester ExoPLAP.

We found a substantial TRAIL expression in the ExoPLAP from the first trimesters and their gradual reduction in the second trimester and third trimester. High expression of these genes in the first-trimester ExoPLAP may indicate the physiological need to develop tolerance toward the developing fetus (33), which could have been an important implication to pregnancy immunotolerance and fetal protection from viral infection (34).

Besides, TIMP-1, an endogenous inhibitor of MMPs, is associated with the integrity of the fetal layer until labor (35). Decreased TIMP-1 levels in the amniotic fluid with enhancing gestation period resulted in a marked increase in MMP-9 before the labor onset (36). We found a higher transcript abundance of TIMP-1 in the first trimester of ExoPLAP.

Toward the end of the first trimester (between 10 and 12 weeks of gestation), the fully established maternal intra-placental circulation led to a burst of oxidative stress in the placenta. Mitochondrial-encoded oxidative phosphorylation genes were associated with the oxidative stress challenged IUGR placentas (37). Excessive placental oxidative stress could be a factor of early pregnancy failure and preeclampsia in later stages. Thus, the gradual reduction in the expression of oxidative stress-associated genes, such as *ND5* between the first and second trimester in ExoPLAP observed in our study may indicate a prerequisite adaptation of the placenta to avoid excessive oxidative stress response. ND5 protein is a subunit of NADH dehydrogenase (ubiquinone) in the mitochondrial inner membrane. Besides, *ND5*, reduced expression of a few other genes associated with oxidative stress signaling gene pathway, such as NADH:ubiquinone oxidoreductase (NDUFs) and *TNFSF10* (38), was observed in the second-trimester ExoPLAP relative to the first trimester.

Unlike the above genes, transcript levels of a few of the identified genes were found to increase toward the late second or third-trimester ExoPLAP, such as *NEDD1, S15, ATF6*, and *NRF1. NEDD1*Neural precursor cell expressed developmentally downregulated protein1 (*NEDD1*) plays a vital role in proper cell positioning (39, 40). The other gene, *ADCY5*, is one of the most abundant members of the adenylate cyclase family. It plays a role in uterine quiescence and labor initiation in mice (41). Although its significance in human placenta development is still unclear, a recent study associated *ADCY5* gene polymorphism with glucose metabolism during pregnancies in non-European descent populations (42). Cyclic AMP-dependent transcription factor *ATF6* was another gene that had low abundance in the first trimester ExoPLAP. The probable reason for its reduced levels is to avoid vascular complications due to excessive ER stress and impaired nitric oxide pathway in early pregnancy (43).

During early placental development, genetic input for fetal growth, through the process of genomic imprinting, is an essential determinant of severe pregnancy complications, such as fetal growth restriction. Thus, we determined the temporal expression of a few imprinted genes in ExoPLAP. Evaluating the parent of origin was, however, beyond the scope of this study.

We detected the expression of imprinted genes in ExoPLAP mRNA levels; all the imprinted genes that we studied, except *IGF2*, were upregulated in the first trimester-ExoPLAP. *M*aternally expressed *PHLDA2* and *CDKN1C* genes are located on the centromeric domain of the Chr11p15 imprinting cluster. *PHLDA2* encodes a 144 amino acid long protein with a Pleckstrin-homology (PH) domain and binds with membrane phosphatidylinositol phosphate lipids (44). It acts as a cell-signaling protein and a negative growth suppressor, useful to predict birth weight (44, 45). In comparison, *CDKN1C* encodes a cyclin-dependent kinase inhibitor that negatively regulates cell proliferation and is highly expressed in P-TGCs, glycogen cells, fetal endothelium, syncytiotrophoblast (ST), and some larger S-TGC nuclei dynamically during mid-to-late placental development (46). The *DLK1* gene is, however, a paternally expressed gene located in the human chromosome 14q32 imprinting cluster. *DLK1* encodes a transmembrane glycoprotein with six epidermal growth factors like repeat motifs, known to act as a growth promoter and is involved in adipogenesis (47, 48). The rest of the imprinted genes that we studied could come from either parent; these include *GRB10, MEG3, PEG 10, IGF2*, and *H19*. *GRB10* is expressed in the fetal endothelium. The maternal inheritance of a disrupted *GRB10* (growth factor receptor-bound protein 10) allele results in placental and embryonic overgrowth (49).

In contrast, the paternal transmission of a disrupted *GRB10* is associated with placental and embryonic growth retardation that persists into adulthood (50). Similarly, maternal inheritance of the Meg3 deletion gene has been associated with loss of imprinting (LOI) of adjacent genes. It results in neonatal death but with apparently normal placental development (51).

While paternal inheritance of the same *MEG3* deletion was associated with impaired fetal and placental growth, the *PEG 10* gene was also found in higher abundance in the first trimester ExoPLAP. Its paternal inheritance results in relatively normal placental development until ~E8.5 in mice (52).

In addition, we studied one more pair of interesting, imprinted genes, *IGF2* and *H19*, as they have been reported to have a reciprocal expression in the placenta. *IGF2* and *H19* imprinted gene clusters are located on human chromosome 11p15. Their reciprocal imprinting is controlled by differential methylation of imprinting control region 1 (ICR1), which is normally only methylated on the paternal allele (48). The unmethylated maternal ICR1 allows the binding of the CTCF transcription factor, blocking the access of *IGF2* promoters to the *H19* downstream enhancers, resulting in the activation of *H19* expression. Decreased *IGF2* expression in the placenta has been associated with growth-restricted SRS cases (53). Our study found a significant rise in fold expression of *H19* at the first trimester relative to the second or third trimester. In contrast, *IGF2* had higher expression in the second trimester than the other two trimesters ExoPLAP.

## Conclusion

We have, for the first time showed a change in ExoPLAP transcriptome with gestation advancement in healthy pregnancies. In addition, we demonstrated the expression of pregnancy-associated placental genes in the ExoPLAP transcriptome. Thus, ExoPLAP in the maternal blood could serve as a non-invasive source of pregnancy-associated placental genes for placental health monitoring during pregnancy. Future studies on the ExoPLAP transcriptome in unhealthy pregnancies are warranted.

## Data Availability Statement

The original contributions presented in the study are included in the article/Supplementary Material, further inquiries can be directed to the corresponding author/s.

## Ethics Statement

The study protocol has been approved by the Human Ethics Committee of Sanjay Gandhi Postgraduate Institute of Medical Sciences. (Ref. no: IEC-2017-9-EMP-95, dated Sept 16, 2019). The patients/participants provided their written informed consent to participate in this study.

## Author Contributions

ST and AP: conceptualizations. ST: funding, project administration, supervision, and visualization. DK, AG, MS, and ST: patient recruitment. AP, MS, SM, DC, DK, AG, and ST: analysis, investigation, and methodology. SM and ST: original draft. All authors contributed to the article and approved the submitted version.

## Funding

This work was supported by the DBT Grants (BT/PR22417/MED/97/348/2016 and BT/PR30361/MED/97/445/2019) to ST.

## Conflict of Interest

The authors declare that the research was conducted in the absence of any commercial or financial relationships that could be construed as a potential conflict of interest.

## Publisher's Note

All claims expressed in this article are solely those of the authors and do not necessarily represent those of their affiliated organizations, or those of the publisher, the editors and the reviewers. Any product that may be evaluated in this article, or claim that may be made by its manufacturer, is not guaranteed or endorsed by the publisher.
