# Supplementary material for: Circulating Placental Alkaline Phosphatase Expressing Exosomes in Maternal Blood Showed Temporal Regulation of Placental Genes
Source: Front Med (Lausanne). 2021 Dec 24;8:758971. doi: 10.3389/fmed.2021.758971 (PMC8739800; doi:10.3389/fmed.2021.758971)
Supplement: Supplementary file 1 [file Table_1.DOCX]

**Supplementary table 1.** List of primers used in the study

| Gene name | Forward (5’-3’) | Reverse (5’-3’) |
| --- | --- | --- |
| H19 | ATCGGTGCCTCAGCGTTCGG | CTGTCCTCGCCGTCACACCG |
| PHLDA2 | GAGCGCACGGGCAAGTA | CAGCGGAAGTCGATCTCCTT |
| PEG10 | GTCGAAGAAACCTGACTGCG | CGCTTATTTCACGCGAGGAC |
| PEG3 | CCCAGACAGGTCCCTGAGTA | GTGGCAGACAAGTGCTTTGG |
| IGF2 | GACCGCGGCTTCTACTTCAG | AAGAACTTGCCCACGGGGTAT |
| GRB10 | CACGAGCCCTTGCTGTTTA | AAGTCCCCTCCACCCTTCTA |
| CDKNIC | CCTGCTGGAAGTCGTAATCC | CACGATGGAGCGTCTTGT |
| MEG3 | CACTGCTTCCTGACTCGCTCTA | TGTGCTTTGGAACCGCAT |
| DLK | GTACTCGGGAAAGGACTGCC | CTCGCAGAAATTGCCTGAGA |
| KCNG4 | AGTTCATCCGCTTCCACCAG | CACTCAGGGAAGCCCTTCAG |
| ADCY5 | CAGAAGCGGAAAGAAGAGAAGC | CCAGAAACTCATCCACTTCATCC |
| NEDD1 | TCCGAAAAGTTTGCCTCGTCT | GTCGCCACTGGAAGATGCTG |
| TNFSF10 | CATCTATTCCCAAACATACTT | CCCTTGATAGATGGAATAGA |
| OR5H6 | CCGCAAGCAGATGACCAAGA | TGGCAAGACATAGACACCTCT |
| ITGAV | GGAGGGAAGCAAAGGACCG | GAGTCCCGAGAGAAGAAGCG |
| APOL3 | CTGGCACCATGGACTCAGAA | GAGAGCATCTGCCTCATCCC |
| KCNH2 | CTGGCAGAGAAACAGCTGGA | CACCCTCCGGTAGTAAAGGC |
| NOS3 | GACTTCACACGCACCACAAA | AGGGTGTGGATCCCGTCTAC |
| TIMP1 | GGCAACTTTGGAGAGGCGAG | CAGCAGCGCTTACCGATGTC |
| TGFbRIII | AAAGAACCGCATGAGCCTGAA | AAGGATTCTCTGCCGTCCCT |
| BLNK | CCTCTTTAGCACGTAACCCTTC | AGGCACCTTGAATGGGCATAG |
| ATF6 | ATCACGGAGTTCCAGGGAGA | AACCTTTAGACGCGAACCCG |
| s15 | GCCGAAAGAAGAGGCAAACC | TGCACATCAAATCTGGGGCT |
| NEDD4L | CAGTGGAGATTTGTGAACAGGG | CTAGAATCCACCCCTTCAAATCCTTG |
| PGC1A | GCTTTCTGGGTGGACTCAAGT | GAGGGCAATCCGTCTTCATCC |
| NRF1 | GCTGATGAAGACTCGCCTTCT | TACATGAGGCCGTTTCCGTTT |
| NRF2 | TCAGCGACGGAAAGAGTATGA | CCACTGGTTTCTGACTGGATGT |
| MFN1 | GGCATCTGTGGCCGAGTT | ATTATGCTAAGTCTCCGCTCCAA |
| OPA1 | GTGCTGCCCGCCTAGAAA | TGACAGGCACCCGTACTCAGT |
| FIS1 | TACGTCCGCGGGTTGCT | CCAGTTCCTTGGCCTGGTT |
| DRP1 | TGGGCGCCGACATCA | GCTCTGCGTTCCCACTACGA |
| ND5 | ACATCTGTACCCACGCCTTC | TCGATGATGTGGTCTTTGGA |
| 18S | GGCCCTGTAATTGGAATGAGTC | CCAAGATCCAACTACGAGCTT |
| IL-10 | GTG ATG CCC CAA GCT GAG A | CAC GGC CTT GCT CTT GTT TT |
| IL-13 | GCATGGTATGGAGCATCAACCTGA | CCTCTGGGTCTTCTCGATGGCA |
| ORC | TTGGCCATGACGATCAGCTT | TGGATGAACACAGCTTGGCT |
| GNAS | AAAGGCGGGAAGAACTTGCT | CTGTCCAGCTTACGGTTGCT |
