# Supplementary figures and images for: Circulating Placental Alkaline Phosphatase Expressing Exosomes in Maternal Blood Showed Temporal Regulation of Placental Genes
Source: Front Med (Lausanne). 2021 Dec 24;8:758971. doi: 10.3389/fmed.2021.758971 (PMC8739800; doi:10.3389/fmed.2021.758971)

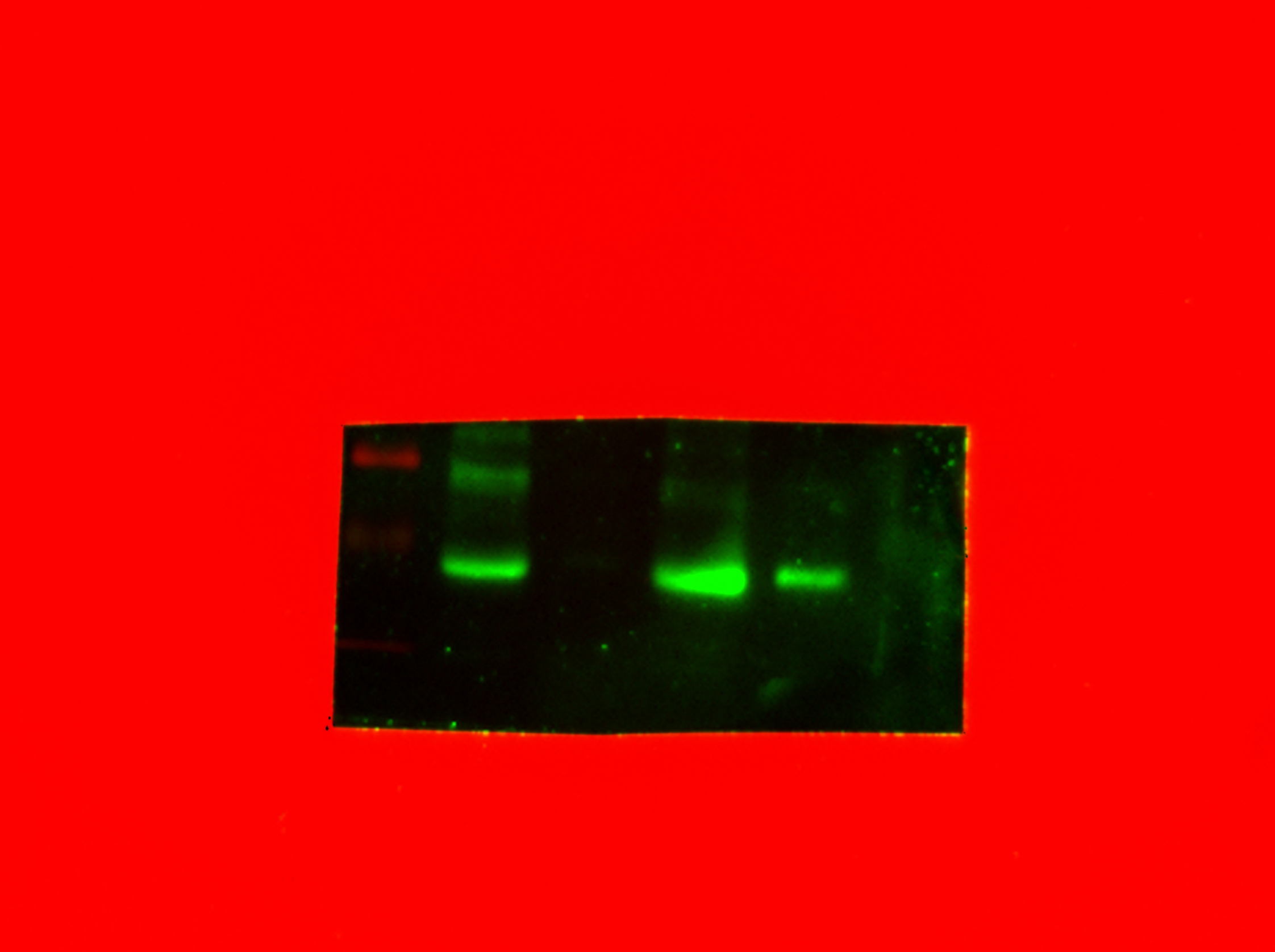

Supplement: Supplementary file 2 [file Image_1.JPEG]

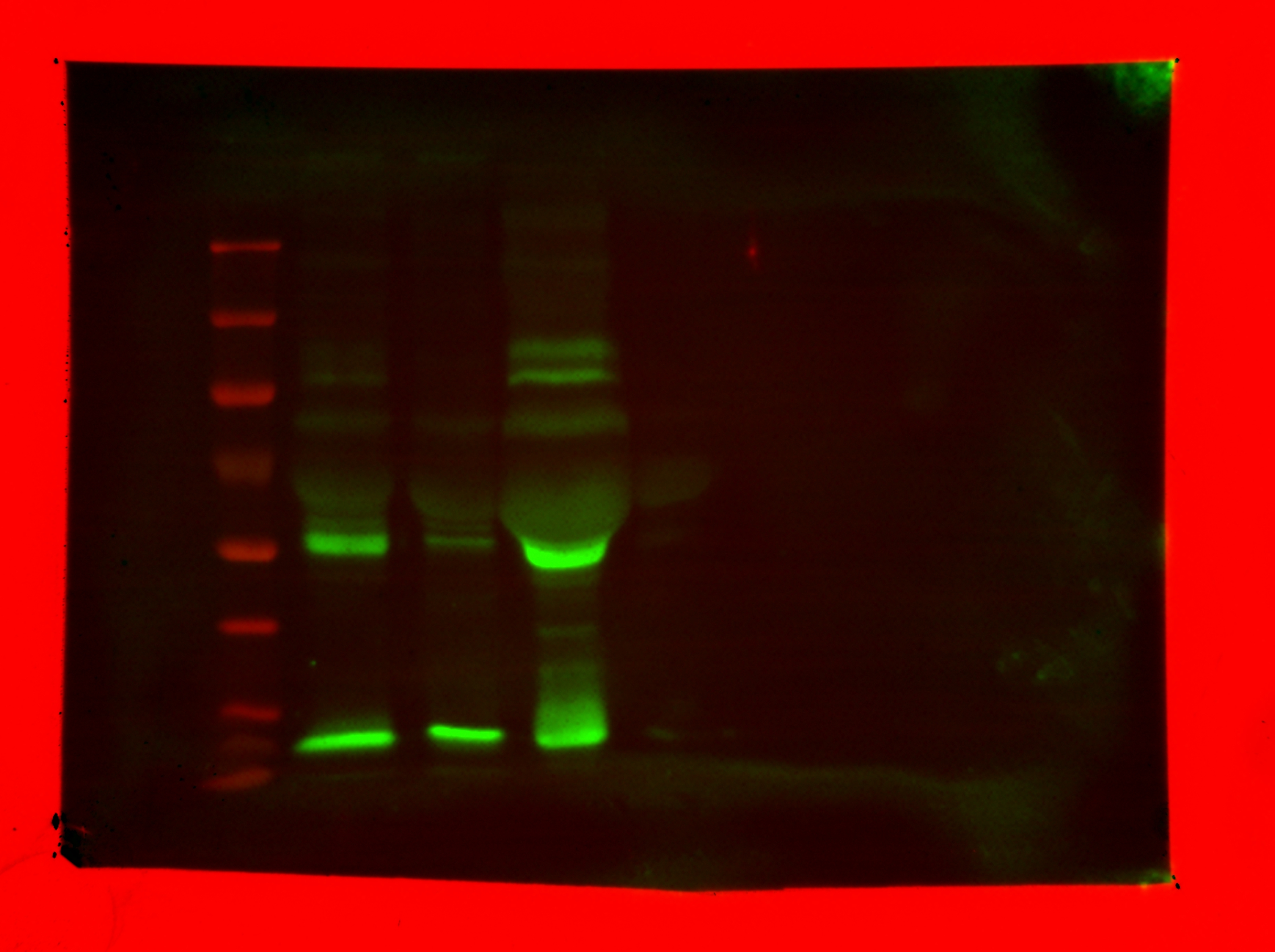

Supplement: Supplementary file 3 [file Image_2.JPEG]
